# Dietary Walnuts Preserve Aspects of Health Span and Alter the Hippocampal Lipidome in Aged High-Fat Diet-Fed Mice

**DOI:** 10.3390/ijms24032314

**Published:** 2023-01-24

**Authors:** Ardijana Novaj, Matthew G. Engel, Ruixuan Wang, Kai Mao, Xiaonan Xue, Yam Amir, Gil Atzmon, Derek M. Huffman

**Affiliations:** 1 Department of Molecular Pharmacology, Albert Einstein College of Medicine, Bronx, NY 10461, USA; 2 Institute for Aging Research, Albert Einstein College of Medicine, Bronx, NY 10461, USA; 3 Department of Epidemiology & Population Health, Albert Einstein College of Medicine, Bronx, NY 10461, USA; 4 Department of Human Biology, University of Haifa, Haifa 3498838, Israel; 5 Department of Medicine, Albert Einstein College of Medicine, Bronx, NY 10461, USA; 6 Department of Genetics, Albert Einstein College of Medicine, Bronx, NY 10461, USA

**Keywords:** diet, aging, nuts, health

## Abstract

Evidence continues to accrue that aging and its diseases can be delayed by pharmacologic and dietary strategies that target the underlying hallmarks of the aging process. However, identifying simple, safe, and effective dietary strategies involving the incorporation of whole foods that may confer some protection against the aging process is also needed. Recent observational studies have suggested that nut consumption can reduce mortality risk in humans. Among these, walnuts are particularly intriguing, given their high content of n-3 fatty acids, fiber, and antioxidant and anti-inflammatory compounds. To this end, 12-month-old male CB6F1 mice were provided either a defined control low-fat diet (LFD), a control high-fat diet (HFD), or an isocaloric HFD containing 7.67% walnuts by weight (HFD + W), and measures of healthspan and related biochemical markers (*n* = 10–19 per group) as well as survival (*n* = 20 per group) were monitored. Mice provided the HFD or HFD + W demonstrated marked weight gain, but walnuts lowered baseline glucose (*p* < 0.05) and tended to temper the effects of HFD on liver weight gain (*p* < 0.05) and insulin tolerance (*p* = 0.1). Additional assays suggested a beneficial effect on some indicators of health with walnut supplementation, including preservation of exercise capacity and improved short-term working memory, as determined by Y maze (*p* = 0.02). However, no effect was observed via any diet on inflammatory markers, antioxidant capacity, or survival (*p* = 0.2). Ingenuity Pathway Analysis of the hippocampal transcriptome identified two processes predicted to be affected by walnuts and potentially linked to cognitive function, including estrogen signaling and lipid metabolism, with changes in the latter confirmed by lipidomic analysis. In summary, while walnuts did not significantly improve survival on a HFD, they tended to preserve features of healthspan in the context of a metabolic stressor with aging.

## 1. Introduction

Aging is characterized by a functional decline in multiple cellular processes and organ systems, including those relevant to metabolic, cardiovascular, and cognitive health, which ultimately culminates in chronic disease, frailty, and death [1]. However, several dietary and pharmacologic interventions have been shown capable of delaying the onset of age-related diseases, functional decline, and extending lifespan in model organisms by targeting underlying hallmarks of the aging process [2,3,4]. Among these, the most well-cited example, dietary restriction (DR), can profoundly delay disease development and extend lifespan in diverse species [1]. However, more recent nutritional investigations focusing on factors beyond a reduction in calories *per se*, have yielded new insights, by either manipulating meal timing via intermittent fasting [5,6], the composition of macronutrients [7,8], or specific dietary components, such as branched-chain amino acids (BCAA) [9] or methionine [10,11,12], thereby revealing marked improvements in health and/or lifespan. 

Several studies have also investigated the ability to supplement intake with single dietary constituents at high doses as a means of conferring metabolic, antioxidant, and/or anti-inflammatory benefits to improve health and lifespan. Indeed, antioxidant supplementation has long been speculated as a means of conferring such benefits, but human and animal data have produced largely equivocal and somewhat conflicting results in this regard [13,14,15,16]. However, the seminal observation that resveratrol, a polyphenol found in grape-seed extracts could improve health and survival in mice on a high-fat diet (HFD) [17] boosted prospects that such strategies could potentially be harnessed to succeed as interventions. However, a follow-up study in the NIA-supported Intervention Testing Program (ITP) did not observe a significant effect of resveratrol on lifespan when provided to mice on a standard chow diet [18]. Likewise, many other dietary components have failed to confer improvements in lifespan as supplements on a standard diet, including green tea extract, curcumin, medium-chain triglyceride oil, and fish oil [18]. However, a limited number of successes have been reported with single dietary compounds on rodent lifespan, including high-dose glycine in ITP [19], as well as supplementation with polyamine spermidine [20]. 

Another approach to leveraging dietary strategies involves the intake of specific whole foods with the potential to confer benefits against age-related risk factors, disease, and mortality. To this end, consumption of coffee [21] as well as total nut consumption [22], have been associated with a reduction in total and cause-specific mortality. Regarding the link between tree nut consumption and mortality, the potential role of walnuts is specifically intriguing given their link to reduced risk for developing many diseases of aging, including type 2 diabetes [23], cardiovascular disease [24], cognitive decline [25], and certain cancers [26]. Moreover, a potential advantage of whole walnuts is that they are uniquely enriched in many bioactive constituents purported to have protective effects, including polyunsaturated fatty acids (e.g., ALA), ᵧ-tocopherol, phytosterols, several polyphenolic compounds, and fiber [26,27,28], of which similar compounds have been previously tested for aging effects as single agents. However, to what extent walnuts per se, could confer potential benefits on health span or life span has not yet been tested. 

Thus, we aimed to perform a careful evaluation of a walnut-enriched diet on healthy aging and longevity in male mice at a dose comparable to human trials, which we and others have shown can confer benefits in rodents [29,30]. Moreover, resveratrol, which is also a plant-derived polyphenol, improved survival in mice under the stress of a purified HFD [17], but not in mice when incorporated into standard grain-based chow formula [31], which are also known to be high in fiber, phytoestrogens and other healthy components. Likewise, our prior observations with walnuts on intestinal tumor development demonstrated favorable effects mainly in obese male mice [30]; thus, we evaluated the ability of walnuts, when introduced in middle age, to delay features of aging and improve survival in the context of an HFD, in a well-established male hybrid rodent model of aging.

## 2. Results

### 2.1. Walnuts Tend to Preserve Insulin Action and Prevent Increased Liver Weight Accrual on an HFD

**Figure 1:**
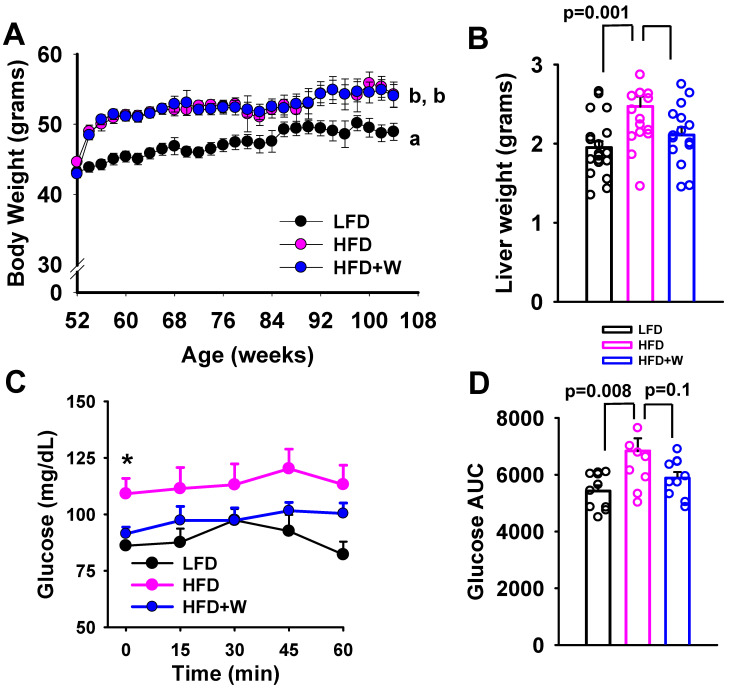
Effect of dietary walnuts on body weight and insulin sensitivity in HFD-fed male CB6F1 mice. (**A**) Male CB6F1 mice demonstrated similar weight gain over 1 year when placed on either the HFD or HFD supplemented with 7.67% walnuts (HFD + W) at 12 mo of age, which was significantly heavier than LFD controls (*n* = 15 per group). (**B**) Excised liver weights following ~7 mo on diet at 19 mo of age (*n* = 16–19 per group). (**C**,**D**) Insulin tolerance tests were also performed (1 mU/kg) at ~18 mo of age and blood glucose levels were monitored over 60 min after injection (*n* = 10 per group). While all groups of aged CB6F1 mice demonstrated marked insulin resistance, as demonstrated by a failure of insulin to suppress glucose levels, levels remained most elevated in HFD-fed mice, which is reflected in a significantly higher glucose area under the curve (AUC). Line and bar graphs represent mean ± SE. * Significantly different from LFD and HFD + W, *p* ≤ 0.05. Dot plots overlaid on bar graphs represent individual data points. Different letters denote a significant difference between groups by Tukey HSD, *p* ≤ 0.05.

At 12 mo of age, mice were assigned to receive either the LFD, HFD, or HFD + W, and body weight was monitored every 2 weeks throughout the study. Despite the fact that mice were middle-aged, groups switched to either of the HFD formulas, which were more energy dense than the LFD (4.6 Kcal/g vs. 3.6 Kcal/g), still demonstrated a marked and rapid weight gain of >23% over baseline within weeks of introduction (Figure 1A). Meanwhile, mice switched from regular chow to the LFD formula also demonstrated a sustained, albeit slower weight gain over this same time period, and remained lighter than HFD and HFD + W groups (Figure 1A). Interestingly, while HFD increased liver weight after ~8 mo on diet, presumably due to fat accrual, this increase was not observed in HFD + W mice, in spite of similar weight gain in these groups (*p* < 0.05; Figure 1B). 

To characterize the effects on insulin sensitivity, we performed an ITT in random-fed mice by injecting insulin at 1 mU/kg and monitoring glucose levels over 1 h (Figure 1B,C). At baseline, glucose levels were lower in LFD and HFD + W-fed groups, as compared to HFD (*p* < 0.05; Figure 1C). However, despite injecting a high dose of insulin, a relative inability to acutely suppress glucose was observed in all groups of aged animals, though levels were persistently highest in HFD mice, which was lowest in the LFD group (*p* = 0.008; Figure 1D) and tended to be lower in HFD + W mice (*p* = 0.10; Figure 1D). Meanwhile, when assessing metabolic signaling in the liver under basal, unstimulated conditions, we observed a slight, but non-significant increase in pAkt in high-fat fed groups, but phosphorylated and total levels of Akt, S6, Erk and AMPK were otherwise unaffected by diet under these conditions (Figure S1A,B).

### 2.2. Walnuts Preserve Working Memory with Aging, but No Significant Effect on Frailty or Survival

**Figure 2:**
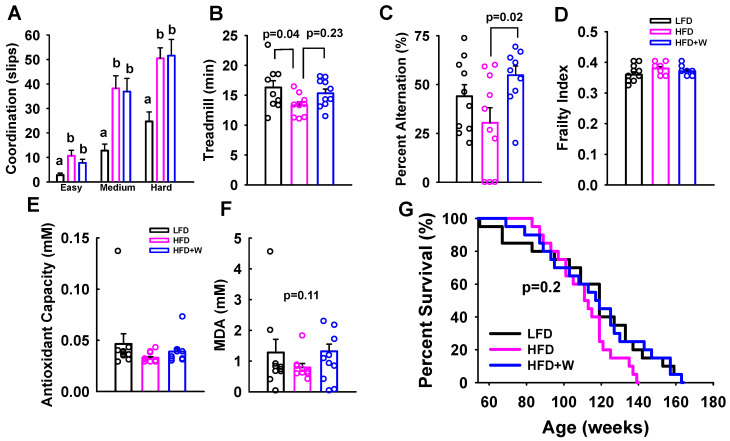
Effect of dietary walnuts on healthspan and lifespan in HFD-fed male CB6F1 mice. (**A**) When subjected to balance beam, LFD animals demonstrated the fewest number of slips on all three beams of varying difficulty, while the number of slips was similarly and significantly increased in both cohorts of HFD-fed animals (*n* = 10 per group for all tests) (**B**) Exercise capacity was also evaluated using a modified motorized treadmill, and revealed that voluntary exhaustion was significantly worsened in HFD mice, as compared to LFD (*p* = 0.04), though exercise capacity tended to be greater in HFD + W versus HFD (*p* = 0.23 after Tukey adjustment), and was comparable to LFD performance (*p* = 0.73). (**C**) Y maze was further used to assess short-term working memory, and while percent alternations tended to decline in HFD versus LFD mice, HFD + W animals demonstrated a marked and significant improvement in working memory versus HFD controls (*p* = 0.02). (**D**) Frailty scores, as determined by a 31-point preclinical frailty index, did not detect any significant differences among groups. In plasma, we further assessed antioxidant capacity and the lipid peroxidation marker, (**E**,**F**) MDA, but did not observe any significant differences in these measures among groups. (**G**) Furthermore, no significant overall effect was observed on survival from 12 mo of age (*p* = 0.2) among groups (*n* = 20 per group). Bar graphs represent mean ± SE. Dot plots overlaid on bar graphs represent individual data points. Different letters denote a significant difference between groups after Tukey HSD adjustment. Exact *p*-values for treadmill and Y-maze indicate significant levels between groups with Tukey HSD adjustment, the ANOVA test for MDA, and log-rank test, respectively.

We next evaluated the effect of our dietary interventions on other aspects of healthspan. We have previously demonstrated that gross motor coordination by balance beam performance and endurance exercise capacity by forced treadmill running decline with aging, but can be mitigated via specific treatments [32,33]. To determine if walnut supplementation might preserve coordination with age, we evaluated the number of slips encountered by animals when challenged to cross round beams of increasing difficulty. LFD animals demonstrated the fewest number of slips, and hence best motor coordination across the easy, medium, and hard beam, while the number of slips was similarly and significantly increased in both cohorts of HFD-fed animals (*p* < 0.05; Figure 2A). Using a modified motorized treadmill fatigue protocol that we have previously developed and utilized, time to voluntary exhaustion was significantly worsened in HFD mice, as compared to LFD (*p* = 0.04; Figure 2B). Meanwhile, we observed a numerical increase in exercise capacity fatigue time in HFD + W versus HFD, that while not significant after Tukey adjustment (*p* = 0.23; Figure 2C), was comparable to LFD performance (*p* = 0.73; Figure 2B). 

Strikingly, when short-term working memory was assessed by Y maze, while percent alternations tended to decline in HFD versus LFD mice, HFD + W animals demonstrated a marked and significant improvement in working memory versus HFD controls (*p* = 0.02; Figure 2C). When surveying hippocampal signaling, while a numerical decrease in pErk was observed in the hippocampus of HFD animals, phosphorylated and total levels of Akt, S6, Erk, and AMPK were similarly unaltered among groups (Figure S1C,D). Likewise, markers of AMPA receptors in the hippocampus did not reveal any significant effect on levels of subunits GluA1 or GluA2 (Figure S2A–C) or levels of phosphorylated Tau (Figure S2D).

We further assessed health status via a 31-point preclinical frailty index, which has been shown to be sensitive to changes in overall health status with aging, but we did not detect any significant differences in this index among groups (Figure 2D). Meanwhile, in contrast to previous studies which have demonstrated that walnuts can boost antioxidant capacity and reduce circulating MDA levels, we did not observe any significant differences in these plasma markers among groups (Figure 2E,F). Likewise, no effect of diet was observed on NF-κB signaling pathway and stress-activated protein kinase, JNK, in the liver and hippocampal tissue by Western blotting (Figure S3A,B), while no signal was detected for activated NF-κB in the hippocampus. Likewise, we did not observe any differences in the expression of inflammatory markers, including IL-1β, TNF-α, or IL-6, in the liver, kidney, or cerebral cortex (Figure S3C). However, the ratio of CD4+/CD8+ T cells isolated from the spleen tended to be lower in HFD animals but preserved in HFD + W mice (*p* = 0.08; Figure S4). Moreover, when assessing effects on survival in mice fed one of the three experimental diets beginning at 12 mo of age, despite a modest reduction of ~6 mo in the maximum lifespan of HFD animals versus other groups, there was no significant impact on overall survival, as determined by log ranked test (*p* = 0.20; Figure 2G) or Taron and Ware Test (*p* = 0.40).

### 2.3. IPA Identifies Estrogen Receptor β and Lipid Metabolism as Potential Targets of Walnuts in Hippocampus

**Figure 3:**
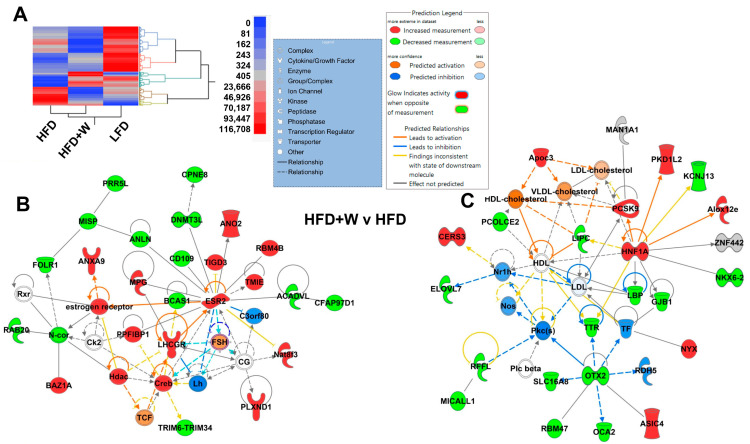
Effect of HFD and dietary walnuts on the hippocampal transcriptome in aged male mice. (**A**) Disparate effects on the hippocampal transcriptome were observed among diets, as can be visualized by the heatmap. A total of 1085 genes reached significance after Tukey HSD, though none reached the genomic threshold of significance after multiple comparison corrections (Table S1). (**B**,**C**) IPA was performed between HFD and HFD + W for each group comparison and intriguingly, two networks of interest emerged, including estrogen signaling, whereby *estrogen receptor* and *ESR2*, were observed to be increased in HFD + W mice, as compared to HFD (*p* = 0.002), and walnuts are highly enriched in phytoestrogens, which have been linked to improved cognitive outcomes. (**C**) Likewise, genes involved in cholesterol metabolism, including *ApoC3* and *PCSK9*, were increased in HFD + W, which were predicted to alter hippocampal HDL, LDL, and VLDL cholesterol handling.

Given our observation that walnut intake preserved working memory in HFD-fed mice, we further interrogated hippocampal tissue for potential clues regarding possible contributors to this effect. To this end, we performed an unbiased assessment for any dietary effects in the hippocampus by RNAseq analysis. Disparate effects on the hippocampal transcriptome were observed among diets, as can be visualized by the heatmap (Figure 3A). Next, using Wilcoxin/Kruskal-Wallis Tests (Rank Sum) and setting the criterion to a minimum of 5 reads, we analyzed 22,310 genes. All pairs were then compared with 1085 genes reaching significance after Tukey HSD (*p* < 0.05), though none reached the genomic threshold of significance after multiple comparison corrections (Table S1). When comparing HFD + W to LFD, the closest genes were ENSMUSG00000097974 (*GM10605*), ENSMUSG00000026173 (*PLCD4*), and ENSMUSG00000097649 (*GM10561*). When comparing HFD to HFD + W, the closest were ENSMUSG00000106176 (*GM43730*), ENSMUSG00000096257 (*CCER2*) and ENSMUSG00000021553 (*SLC28A3*). Finally, when comparing HFD to LFD, ENSMUSG00000019710 (*MRPl24*) and ENSMUSG00000019987 (*ARG1*) were most closely approaching significance. Meanwhile, ENSMUSG00000106176 (*GM43730*) approached the genomic threshold for significance in HFD + W vs. LFD and HFD vs. HFD + W comparisons.

Next, we performed IPA focusing on possible differences between HFD vs. HFD + W treatments, and intriguingly, two networks of interest emerged. The first involves estrogen, where *estrogen receptor*, as well as *ESR2*, which was increased in HFD + W mice, as compared to HFD (*p* = 0.002), were predicted as potentially important nodes of activation by walnuts (Figure 3B). This is particularly intriguing given that walnuts are highly enriched in phytoestrogens, which have been linked to improved cognitive outcomes. Likewise, *ApoC3* and *PCSK9* among others were increased in HFD + W, which were predicted to alter key aspects of hippocampal lipid and specifically cholesterol metabolism.

### 2.4. Walnut Supplementation Alters the Hippocampal Lipidome

**Figure 4:**
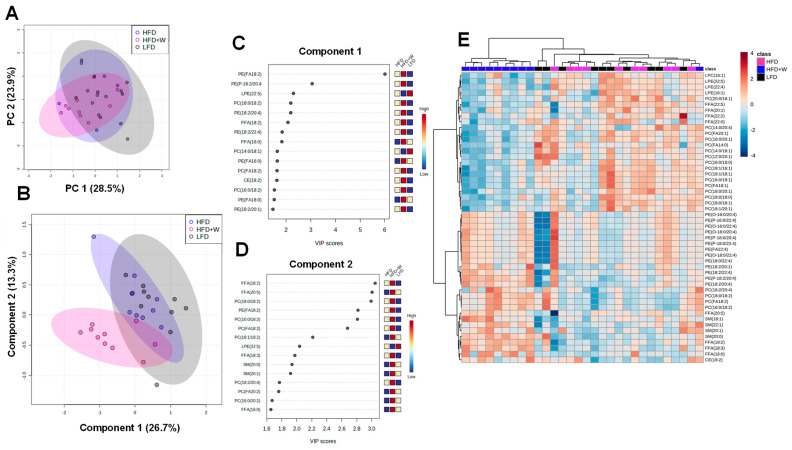
Effect of HFD and dietary walnuts on the hippocampal lipidome in aged male mice. (**A**) When comparing the lipidome among groups by unsupervised principal component analysis (PCA), a high degree of overlap among groups when accounting for all detected lipids was observed. (**B**–**D**) Partial Least Squares Discriminant Analysis (PLS-DA) found that HFD + W mice could be largely distinguished from other experimental groups, driven by species listed in Component 1, particularly PE(FA18:2) and to a lesser extent, species in Component 2, including FFA(18:2), FFA(20:5), PC(18:0/18:2). (**E**) This can be further visualized by a heatmap constrained to 50 lipid species, which detects a high degree of clustering among HFD + W samples.

Given the suggested connection between walnuts and brain lipid metabolism via IPA, including their known enrichment of polyunsaturated fatty acids (PUFAs), we decided to perform targeted lipidomics in hippocampal tissue in 20 mo old mice following 8 mo on diet. After excluding samples with >20% missing data, 252 lipid species were included in the analysis, among which only 16 reached the threshold for significance after FDR correction (Table 1). Interestingly, very few differences were observed between LFD and HFD-fed mice. However, compared to both control groups, HFD supplemented with walnuts (HFD + W) led to a significant reduction in PC(14:0/18:1), PC(16:0/18:0), PC(18:1/16:1), PC(16:0/18:1), PC(FA20:1), PC(FA14:0), PC(FA18:1), LPE (22:5) and FFA(22:5) content, while FFA(18:2), FFA(18:3), SM(20:0), PC(18:0/18:2) were increased. Aligned with this observation, unsupervised principal component analysis (PCA) revealed a high degree of overlap among groups when accounting for all detected lipids (Figure 4A). However, supervised Partial Least Squares Discriminant Analysis (PLS-DA) found that HFD + W mice could be largely distinguished from other experimental groups (Figure 4B), which was mainly attributed to species listed in Component 1, and to a lesser extent, species in Component 2 (Figure 4C,D). This can be further visualized by a heatmap containing 50 top lipid species, which detects a high degree of clustering among HFD + W samples separate from other groups (Figure 4E). Further, when assessing the effects of diet on lipid classes in the hippocampus, HFD + W tended to numerically reduce levels of CER (*p* = 0.07), LPC (*p* = 0.18), and LPE (*p* = 0.11) and CE (*p* = 0.28), while the reduction in PC concentrations was significantly reduced (*p* = 0.02; Table 2).

## 3. Discussion

Accumulating evidence in observational studies suggests that nut consumption, including walnuts, can confer health benefits and reduce mortality risk, though a direct effect on survival has yet to be demonstrated. The initial observations linking walnut intake to health first emerged from observational and clinical trials reporting beneficial effects of moderate walnut consumption on predictors of cardiovascular risk, including favorable effects on cholesterol levels, the lipoprotein profile [34,35], and endothelial function [36]. Further efforts began to uncover a unique and enriched repertoire of bioactive components in walnuts, including ALA, as well as phytochemicals and polyphenolic compounds, conferring a high antioxidant capacity, relative to other foods, with the potential to interfere with tumorigenesis, oxidative stress, and inflammation [37]. Indeed, several studies have now demonstrated that walnut intake can mitigate tumor development in mouse models of breast cancer and intestinal cancer [30,38,39,40]. Effects on the gut are particularly striking, whereby the ability of walnut consumption to potently alter the microbiome may in part confer protection not only from tumorigenesis [38] but also in a dextran sodium sulfate model of inflammatory bowel disease [41]. More recently, walnut consumption has been linked to cognitive benefits to varying degrees in some human observation studies and shown to slow cognitive and behavioral decline in a mouse model of cerebral amyloidosis [42,43,44,45,46].

Given the premise that walnuts contain a plethora of bioactive compounds with potential health benefits, our initial hypothesis was that the favorable effects of walnuts on survival, if present, may be most apparent under conditions of metabolic stress, such as that imposed by an HFD, and as was previously observed with resveratrol, a polyphenolic compound with properties similar to those found in walnuts [17]. Indeed, some prior studies have suggested walnut effects tended to be more pronounced in subgroups at higher risk [44], which is important from a public health perspective, given that a majority of adults in developed countries are now considered overweight or obese. Moreover, as we had previously observed that walnuts were specifically protective from intestinal tumor development in male mice only [30], we reasoned that such protection, if any, would be most likely to occur in males, rather than females. In spite of some favorable signals related to metabolic health, including a reduction in presumed hepatic fat accumulation, as has been observed [47], as well as improved cognition with walnut intake in this study, the effect on overall survival with walnuts, though suggestive, failed to reach significance. 

While it is possible that the detrimental effects of the HFD may have overwhelmed some of the potential benefits of walnut incorporation, the expectation that HFD-fed animals would demonstrate markers of greater inflammation, oxidative stress, and a reduction in lifespan, as compared to LFD mice, also failed to materialize and reach significance. Such an observation appears to be somewhat at odds with prior evidence in high-fat, high-calorie diet-fed mice [17,48]. However, it should be noted that some fundamental differences in our experimental design versus some prior reports could in part explain these differences. First, our HFD-fed animals were compared to mice fed a carefully-matched, purified LFD formula, rather than a poorly-defined chow-based diet. As we and others have highlighted, chow-based formulations contain multiple individual components at levels not seen in defined diets which might be expected to play a role in promoting health and survival independent of their lower digestible energy, including high levels of phytoestrogens, fiber, and Vitamin D [49]. Moreover, while others have demonstrated marked differences in body weight between chow and HFD-fed mice [50], animals provided the LFD at middle age also gained weight, albeit at a slower rate, such that only a peak difference of 10–15% in body mass was achieved between LFD and HFD groups, which narrowed over time. Indeed, ketogenic diets, which are also high in fat, can extend health and life span when caloric intake is restrained and weight gain mitigated, suggesting that a major detriment of these diets is inherent to the relative weight gain and obesity they produce, rather than the high-fat content *per se* [8]. Furthermore, since our intervention was not initiated until middle age (12 mo of age), which we reasoned was most translatable, we cannot rule out that more notable differences may have emerged if the dietary regimens were to be initiated earlier in the lifespan.

A major consideration in interpreting the survival outcome of this study also lies in the major causes of death that occur between humans and rodents. While the strongest evidence linking walnuts to reduced mortality risk in humans includes its favorable effects on cardiovascular risk factors and some aspects of metabolic health [34], normal mice do not develop cardiovascular disease or type 2 diabetes. In contrast, while walnuts have demonstrated the ability to potently mitigate site-specific cancers in genetic mouse models [30,38,39,40], normal-aged mice do not typically develop spontaneous tumors in the breast or gastrointestinal tract or other sites common to humans. Instead, lymphoma is often the most frequently reported malignancy and attributable cause of death in aged mice, and there is no evidence to date to support that walnuts or their constituents can protect against spontaneous lymphoma development and spread in rodents. Thus, these data do not rule out its potential to protect against some leading causes of death in humans, as has been suggested by human observation studies. Such studies addressing this possibility utilizing specific mouse models, such as *ApoE* or *ldlr* mutant mice, which predispose animals to these diseases, with the former being supported by a prior mixed nut intervention [51], warrant further investigation.

Although we failed to observe a significant impact on survival, health span assays suggested a beneficial effect on some indicators of health with walnut supplementation, including improved short-term working memory, as determined by Y maze, and a tendency toward better-preserved glucose homeostasis on an HFD, as determined by a slightly lower baseline glucose and glucose area under the curve during an ITT, as compared to HFD controls. Moreover, exercise capacity tended to be slightly greater in HFD + W versus HFD mice, and was comparable to LFD-fed animals. However, LFD mice demonstrated better gross motor coordination than both high-fat fed groups on the balance beam, which is may be attributed to their slightly smaller body size, while no difference was observed in the frailty index among groups. The observed improvement in working memory was particularly intriguing given the emerging links between walnuts, walnut components, and the central nervous system (CNS), including protection from toxin-induced neuroinflammation [52] and amyloidosis [43], and purported improvements in autophagic function in the hippocampus from aged rats [53]. Intriguingly, IPA identified estrogen signaling as a potentially important node of activation by walnuts in the hippocampus. Indeed, estrogen receptor β signaling via estradiol and phytoestrogen intake has been shown to play an important role in cognition, including executive function [54,55]. This is particularly intriguing given that walnuts, which are highly enriched in phytoestrogens, have also been linked in other reports to improved cognitive outcomes [56,57], though a definitive role of estrogen receptors in this regard would need to be formally tested using more incisive models to confirm. 

A second intriguing pathway that emerged from IPA involved a predicted effect of walnut intake on brain lipid metabolism. Given the known enrichment of polyunsaturated essential fatty acids (PUFAs) in walnuts, we were prompted to examine the lipidome more carefully in the hippocampus. While very few differences were observed between LFD and HFD-fed mice, walnut consumption for ~8 mo most strongly altered aspects of the hippocampal lipidome, including 14 individual lipid species. However, these effects appear to be somewhat complex and were largely driven by reductions in several saturated or monounsaturated containing PC species, as well as LPE (22:5) and FFA (22:5), and overall PC content, with a notable trend in reduced CER levels. Meanwhile FFA(18:2), FFA(18:3), SM(20:0), PC(18:0/18:2) were increased with walnuts. Along these lines, lard and soybean oil were the exclusive lipid sources in the controlled LFD and HFD used in this study, while lard was substituted for an isocaloric percentage of walnuts in HFD + W, with soybean oil concentration held constant [30]. Lard comprises roughly 40% saturated and 60% unsaturated fatty acids, with 11% of total FA deriving from linoleic acid (LA, 18:2n-6) and negligible amounts of alpha-linolenic acid (ALA, 18:3n-3). In contrast, walnuts are a rich source of polyunsaturated fatty acids, with 63% of total FA comprising LA and 13% ALA [58]. The observed increases in FFA 18:2, FFA 18:3, and PC 18:0/18:2 are consistent with hippocampal enrichment with LA and ALA with the addition of dietary walnuts. 

Meanwhile, key long-chain polyunsaturated essential fatty acids (LC-PUFAs), including LPE 22:5 and FFA 22:5 species were suppressed in the hippocampus by walnut intake. Functionally, LC-PUFAs, which include arachidonic acid (AA, 20:4n-6), eicosapentaenoic acid (EPA, 20:5n-3), and docosahexaenoic acid (DHA, 22:6n-3), play vital roles in phospholipid membrane integrity and eicosanoid signaling pathways, and are highly enriched in the CNS [59]. These LC-PUFAs can be synthesized in mammals from the plant-derived 18-carbon precursors LA and ALA, but this process is highly inefficient and subject to feedback regulation and synthetic competition between n-3 and n-6 species [60]. Indeed, the suppression of LPE 22:5 and FFA 22:5 species in HFD + W-fed mice may indicate blockade in an elongation or desaturation step downstream of LA and ALA as a result of increased concentration of these species from the walnut diet. Rodents fed high levels of ALA have been shown cause negligible changes in tissue DHA content [61], consistent with our observations here. 

Walnuts also led to a global reduction in hippocampal PC content, though the physiological implication of this seems somewhat complex and difficult to reconcile with the general assertion that PCs benefit the brain and tend to be reduced in models of stress and AD [62,63]. On the other hand, the trend toward reduced ceramide content (*p* = 0.07), whose levels can otherwise increase as a result of sphingomyelin hydrolysis and, can be elevated via adverse stress [63] is suggestive (but not decisively so) of a beneficial impact of dietary walnuts on neuronal health in the hippocampus. Overall, IPA and lipidomic analysis clearly demonstrate that the fatty acids and other bioactives contained in walnuts, can alter brain lipid composition, and unraveling the implications of these changes may be an interesting area for future investigation.

In summary, consistent with some of the reported health benefits previously attributed to walnuts in preclinical models and humans, we were able to detect the benefits of walnuts to elements of healthspan in aged male mice, though no significant impact on survival was observed in this study. Specifically, health span assays suggested some benefits were conferred with walnut intake on improved fasting glucose, short-term working memory, mitigation of liver weight gain and exercise intolerance on HFD. Follow-up-omic analyses further support that walnuts directly modulate features of hippocampal biology, including estrogen signaling and lipid metabolism and tend to lower CER levels. While the focus of this study was on male mice, due to their reportedly unique susceptibility to metabolic duress in the aging process, and typically greater response to interventions that counter such manifestations, such as insulin resistance and inflammation, an inherent limitation of this study is its inability to address the effects of walnuts on female healthspan, which warrants investigation. Furthermore, given the established evidence linking walnut intake to cardiovascular, metabolic, and cognitive health, their utility as an adjuvant dietary strategy for older humans, particularly in subgroups of higher risk, is an intriguing possibility that should also be pursued.

## 4. Materials and Methods

### 4.1. Generation of Walnut Diets

The diets used for this study were produced by Envigo (Madison, WI, USA) as described previously [30]. Details regarding the diet formulations are shown in Table 3. In brief, freshly shelled walnuts supplied by the California Walnut Commission were provided to Envigo, ground on-site, mixed into the matched formula, vacuum packaged into 1 kg bags, stored at 4 °C, and used within 2–3 wks of opening to maximize freshness. The diets produced were a defined control low-fat diet (LFD) at 3.6 Kcal/g, 69.1% Kcal from (carbohydrate) CHO, 20% Kcal from (protein) PRO, and 10.4% Kcal from Fat, a control high-fat diet (HFD) at 4.6 Kcal/g, 36% Kcal from CHO, 19% Kcal from PRO and 45% Kcal from Fat, and an isocaloric and macronutrient-matched HFD containing 7.67% walnuts by weight (HFD + W). Furthermore, adjustments in micronutrient concentrations were made to the HFD formulas to account for their increased caloric density and expected reduction in food intake by mass, as we have shown previously [30]. Moreover, pellets were routinely replaced with fresh diet in the hopper twice per week. All experimental methods were approved by the IACUC at the Albert Einstein College of Medicine under protocols 20150103 (26 January 2015), 20170814 (17 November 2017), and 00001273 (17 September 2020).

### 4.2. Animals and Design

CB6F1 male mice were obtained from the NIA and assigned to one of three dietary groups for life span studies (*n* = 20 per group) or health span and other interim analyses at 19–20 mo of age (*n* = 18–19 per group). We elected to only focus on male mice here, due to their reportedly unique susceptibility to metabolic duress in the aging process, and greater response to interventions that counter manifestations of metabolic duress, such as insulin resistance and inflammation, such as 17α-estradiol, as well as anti-diabetic and anti-inflammatory agents [2,64,65,66,67]. Animals deemed severely moribund and anticipated to not survive another 48 hrs were immediately euthanized and this was considered the time of death. Animals were group housed 4 per cage under a standard light/dark photoperiod 14L:10D at 22 °C and provided food and water *ad libitum*. 

### 4.3. Basic Physiology Characteristics

To evaluate phenotypic changes, we longitudinally monitored body weight in all cohorts. Insulin sensitivity was assessed by insulin tolerance tests (ITTs) at approximately 19 mo of age. In brief, mice fasted early in the morning for approximately 1 hr, and a baseline blood glucose measurement was made. Animals were then injected IP with insulin (1 mU/kg) and blood glucose levels were checked at 15, 30, 45, and 60 min after injection, as described [68]. At sacrifice, whole livers were collected and weighed.

### 4.4. Physical Performance

Beginning at 18–19 mo of age, assays for physical and cognitive performance were carried out in mice as described in a blinded fashion [32,69]. For gross motor coordination, we employed the balance beam test. In brief, animals were first familiarized with the testing setup by walking twice across a 4ft plank. Animals were then challenged to traverse a 48” long round beam of decreasing difficulty (0.5” difficult, 0.75” medium, 1” easy), with light and food cues as motivation to cross, and the number of slips was counted while crossing the beam. Endurance was determined by a single test on a treadmill (Exer 3/6, Columbus Instruments). In brief, mice were first familiarized with the treadmill for 3 non-consecutive days for 5 min at a walking speed (8 m/min). Animals were then challenged with a graduated fatigue test, beginning at a 4% incline and 8 m/min for 3 min. The protocol used increased speed to 10 m/min at 3 min, 12 m/min at 4 min, and 15 m/min at 5 min and maintained this speed until voluntary fatigue (all mice fatigued prior to 30 min). The frailty of mice at 19 mo of age was determined using a 31-point index previously described using a mouse clinical frailty index as previously described [70]. 

### 4.5. Memory Assessment via Y Maze

Y Maze Spontaneous Alternation is a behavioral test for measuring the willingness of rodents to explore new environments, which can detect deficits in spatial working memory in aged animals [70]. Rodents typically prefer to investigate a new arm of the maze rather than return to one that was previously visited. Testing occurs in a Y-shaped maze. In brief, 18–19 mo old animals were placed in one of the arms, and their spontaneous entries into different arms were counted for 5 min. The percent alternation was calculated as: [number of alternations/(total number of entries − 2)] × 100. Over the course of multiple arm entries, the subject should show a tendency to enter a less recently visited arm. 

### 4.6. Protein Isolation and Western Blotting

Western blotting was performed similarly as described [70]. In brief, tissues were homogenized in RIPA buffer and extracted protein concentration was determined using the BCA protein assay (Sigma, St. Louis, MO, USA). For electrophoresis, 20 μg of total protein was separated on Criterion TGX Stain-Free gels (4–20%, Bio-Rad, Hercules, CA, USA) at 120 V constant for 90 min. Stain-free gels were then imaged prior to transfer on a Bio-Rad Chemidoc MP Imaging System (Bio-Rad, Hercules, CA, USA) to confirm equal protein loading. Gels were then wet transferred onto PVDF membranes at 100 V constant for 1 h, and the equal transfer was confirmed by Ponceau S stain as described. Membranes were then blocked in 5% milk in TBST for 1 h at room temperature and then incubated overnight at 4 °C with primary antibodies from Cell Signaling against p-AktThr308 (1:1000; no. 13038), total Akt (1:1000; no. 4691), p-p44/42MAPKThr202/Tyr204 (1:1000; no. 9101), total p44/42 MAPK (1:1000; no. 4695), p-S6 (1:1000; no. 5364), total S6 (1:1000; no. 2217), total GluA1 (1:1000; #13185), and total GluA2 (1:1000; #5306), pTau (1:1000), p-p65 (Ser536; 1:1000, #3033), total p65 (1:1000; #8242), pIĶĶα/β (1:1000; #2697), IĶĶα (1:1000; #11390), pJNK (1:1000; #9255) and total JNK (1:1000; #9252). Following a 1-h incubation with the appropriate secondary antibody, Clarity Western ECL Substrate (Bio-Rad, Hercules, CA, USA) was applied to the membrane, and bands were visualized using a Bio-Rad Chemidoc MP bioimager to first-pixel saturation. Densitometry was then performed using Image Lab software v5.0 (Bio-Rad, Hercules, CA, USA).

### 4.7. RNA Isolation and RT-qPCR

Total RNA from frozen tissues (liver, cortex, kidney) was isolated using TRIzol^®^ Reagent per the manufacturer’s instructions (Thermo Fisher; Waltham, MA) and quantified via Qubit assay as we have described previously [30,33,71,72,73]. First-strand complementary DNA (cDNA) was synthesized with random primers using Bio-Rad iScript cDNA Synthesis Kit. All qPCR reactions were then carried out using Bio-Rad SsoAdvanced SYBR Green mix on a Bio-Rad CFX384 qRT-PCR Machine. Gene expression was carried out in the liver, kidney, and cortex for *IL6* (For—5′ AGTTGCCTTCTTGGGACTGA and Rev—5′ TCCACGATTTCCCAGAGAAC) *TNF-α* (For—5′ ATGAGAAGTTCCCAAATGGC and Rev—CTCCACTTGGTGGTTTGCTA) and *IL-1β* (For—5′ GCCCATCCTCTGTGACTCAT and Rev—5′ AGGCCACAGGTATTTTGTCG), and all data were normalized to Peptidylprolyl isomerase A (*PPIA*; For- 5′ GCGTCTSCTTCGAGCTGTT and Rev—5′ RAAGTCACCACCCTGGCA) using the ∆∆Ct method. 

### 4.8. RNAseq and Analysis

RNA isolated from frozen hippocampal tissue was isolated using the Trizol^®^ procedure, quantified via Qubit assay, and submitted for Bioanalyzer analysis in the Einstein Genomics Core to confirm quality (RIN score > 9). RNA samples were then submitted to Novogene for RNA sequencing using standard approaches. In brief, after passing confirmatory quality control steps at Novogene, mRNA was then used for library construction and subsequently analyzed via Illumina sequencer (Novaseq 6000 platform, PE150 sequencing strategy; 20 M paired reads). We then used two different strategies for whole genome expression analysis to explore candidate genes associated with the treatment and for pathway analysis (IPA software by Qiagen). As a first approach, genome alignment was performed on clean FASTQ sequence reads received from Novogene after quality control. For genome alignment, the reads were mapped to the mouse reference genome GRCm38 (mm10) with the RNA-seq aligner STAR [74]. In order to estimate gene expression levels, the aligned and annotated reads were then quantified with the featureCounts function of Subread [75]. The quantified reads were normalized into FPKM (Fragments Per Kilobase Million) with the fpkm function of the R package DESeq2 [76]. As a second approach, transcriptome alignment was performed on clean FASTQ sequence reads received from Novogene after quality control. In order to estimate gene expression levels, the reads were mapped to the mouse reference transcriptome (gencode.vM23) and quantified with Salmon [77], then reported in TPM (Transcripts Per Million). Differential expression analysis was completed with DESeq2 [76].

### 4.9. Ingenuity Pathway Analysis

We assembled 3 lists for each comparison (HFDW vs. HFD, HFDW vs. LFD, and HFD vs. LFD) of the top 1000 reads (representing 1000 transcriptions of unknown genes and genes) that were numerically associated (*p* < 0.05) with the diet. These lists were annotated by Ingenuity Pathway Analysis (IPA) and SeattleSeqAnnotation and were used in the IPA analysis v21.0 (www.ingenuity.com) accessed on 20 March 2022. The resulting classification of networks, pathways, biological processes, and molecular functions are represented in graphic format.

### 4.10. Antioxidant Capacity and Oxidative Stress Markers

Total antioxidant capacity in plasma was assayed using the Antioxidant Capacity Assay (Cayman Chem, Ann Arbor, MI, USA) which relies on the ability of antioxidants in the sample to inhibit the oxidation of ABTS^®^ (2,2′-azino-di-[3-ethylbenzthiazoline sulphonate]) to ABTS^®^ · + by metmyoglobin [78,79,80,81]. The capacity of the antioxidants in the sample to prevent ABTS oxidation is compared with that of Trolox, a water-soluble tocopherol analog, and is quantified as molar Trolox equivalents. Oxidative stress was determined by measuring Thiobarbituric Acid Reactive Substances (TBARS) in plasma via the Cayman’s TBARS Assay kit to determine lipid peroxidation (Cayman Chem, Ann Arbor, MI, USA). 

### 4.11. Hippocampal Lipidomics

Lipidomics in hippocampal tissue (~15 mg) was performed by the Northwest Metabolomics Research Center, similar to that which was previously described [32,69,82]. Frozen tissue was first homogenized in water, and samples were subjected to a dichloromethane extraction. Isotope-labeled internal standards mixture (Sciex; Framingham, MA, USA) was then added to each sample before proceeding with additional incubation and centrifugation steps. Extracts were then concentrated under nitrogen and reconstituted in 250 μL of the running solution (10 mM ammonium acetate in 50:50 methanol:dichloromethane). Quantitative lipidomics was then performed with the Sciex Lipidyzer platform consisting of Shimadzu Nexera X2 LC-30AD pumps, a Shimadzu Nexera X2 SIL-30AC autosampler, and a Sciex QTRAP^®^ 5500 mass spectrometer equipped with SelexION^®^ for differential mobility spectrometry (DMS), as described. Notably, 1-propanol was used as the chemical modifier for the DMS. Samples were introduced to the mass spectrometer by flow injection analysis at 8 μL/min. The lipid molecular species were measured using multiple reaction monitoring (MRM) and positive/negative polarity switching. Positive ion mode detected lipid classes SM/DAG/CE/CER/DCER/HCER/DCER/TAG and negative ion mode detected lipid classes LPE/LPC/PC/PE/FFA. Data acquisition and processing were performed using Analyst 1.6.3 and Lipidomics Workflow Manager 1.0.5.0, and processing, normalization, and analysis were performed via MetaboAnalyst 4.0. 

### 4.12. Splenocyte Isolation and Flow Cytometry

Spleens were harvested, homogenized, and passed through a 70 um strainer to collect splenocytes in a falcon tube containing PBS on ice. Cells were then pelleted and resuspended with 1X red blood cell (RBC) lysis buffer and incubated on ice for 5 min. After washing with PBS, 1 million cells were counted and resuspended in PBS. Live cells were separated from dead cells and Fc block was performed subsequently using anti-mouse CD16/32 (Biolegend, San Diego, CA, USA, cat#101301). Cells were then resuspended in FACS buffer (PBS + 2%FBS + 0.2mMEDTA) incubated with cell surface primary antibodies against CD45 (Biolegend, cat#109822), CD4 (Biolegend, cat#100421), and CD8 (Biolegend, cat#100725) on ice protected from light for 30 min. After washing off the antibodies, cells were fixed with 2% PFA, sorted on an LSRII flow cytometer, Becton Dickinson Inc, and data were analyzed using FlowJo software 5.4+. 

### 4.13. Statistics

Cross-sectional data were analyzed by ANOVA, while longitudinal measures were assessed by repeated measures ANOVA, and planned contrasts were performed as appropriate. When a significant main effect was detected, planned two-group contrasts with Tukey Honest Significant Difference [HSD] adjustment were applied. Survival analysis was performed by the Kaplan Meier and log-rank tests as well as the Taron Ware test. All data were log-transformed to ensure normality of distribution and analyses using SPSS (SPSS Inc., Chicago, IL, USA) or statistical software R4.2. All values reported here are means ± standard error (SE). A *p* ≤ 0.05 was considered to be statistically significant.

## Figures and Tables

**Table 1:** Concentration of lipid species in hippocampus.

Species [nmol/g]	LFD	HFD	HFD+Walnut	FDR-Corrected *p* Value
PE(P-18:2/20:4)	3.84 ± 0.20 ^a^	5.38 ± 0.56 ^b^	6.58 ± 0.26 ^b^	<0.001
LPE(22:5)	2.22 ± 0.18 ^a^	2.12 ± 0.16 ^a^	1.29 ± 0.13 ^b^	0.007
PC(18:1/16:1)	169.1 ± 9.9 ^a^	154.6 ± 4.8 ^a^	119.6 ± 7.8 ^b^	0.007
PE(18:2/20:4)	4.06 ± 0.24 ^a^	5.18 ± 0.48 ^b^	5.77 ± 0.18 ^b^	0.007
FFA(18:2)	50.8 ± 4.2 ^a^	52.8 ± 2.7 ^a^	69.4 ± 3.6 ^b^	0.007
PC(14:0/18:1)	163.1 ± 12.4 ^a^	160.2 ± 11.8 ^a^	107.9 ± 9.0 ^b^	0.007
SM(20:0)	184.6 ± 13.1 ^ab^	171.5 ± 4.0 ^a^	204.0 ± 8.2 ^b^	0.008
FFA(18:3)	3.30 ± 0.20 ^ab^	3.18 ± 0.12 ^a^	3.82 ± 0.16 ^b^	0.008
PC(18:0/18:2)	101.9 ± 13.1 ^a^	106.3 ± 8.1 ^a^	141.2 ± 12.9 ^b^	0.022
PC(18:1/18:1)	795.7 ± 46.5 ^a^	769.1 ± 24.9 ^a^	603.9 ± 40.2 ^b^	0.025
FFA(22:5)	38.6 ± 2.0 ^a^	35.5 ± 1.63 ^a^	26.6 ± 1.9 ^b^	0.025
PC(16:0/18:1)	13,783 ± 733 ^a^	13,300 ± 402 ^a^	10,736 ± 623 ^b^	0.031
PC(FA18:1)	20,362 ± 1056 ^a^	19,891 ± 602 ^a^	16,098 ± 924 ^b^	0.041
PC(FA14:0)	266.9 ± 19.9 ^a^	272.2 ± 14.8 ^a^	204.0 ± 8.2 ^b^	0.041
PC(FA20:1)	331.0 ± 18.6 ^a^	322.4 ± 13.9 ^a^	253.2 ± 17.3 ^b^	0.041
PC(16:0/18:0)	771.7 ± 42.8 ^a^	734.7 ± 25.1 ^a^	594.0 ± 30.0 ^b^	0.041

Among 252 detected lipid species in hippocampus, a total of 16 were significantly altered by one or more experimental diets after FDR correction. Different letters denote a significant difference between groups, *p* < 0.05.

**Table 2:** Concentration of lipid classes in hippocampus.

Lipid Class [nmol/g]	LFD	HFD	HFD+Walnut	ANOVA *p* Value
Cholesterol ester (CE)	104.5 ± 7.1	108.1 ± 5.8	93.3 ± 7.1	0.28
Ceramide (CER)	2386 ± 129	2750 ± 219	2124 ± 593	0.07
Free Fatty Acid (FFA)	8751 ± 388	8662 ± 468	9074 ± 371	0.76
Lysophosphatidylcholine (LPC)	840.1 ± 57.5	802.0 ± 27.0	703.2 ± 64.7	0.18
Lysophosphatidylethanolamine (LPE)	308.2 ± 21.4	295.7 ± 17.0	247.2 ± 24.1	0.11
Phosphatidylcholine (PC)	29,982 ± 1227 ^a^	30,171 ± 778 ^a^	26,105 ± 1421 ^b^	0.02
Phosphatidylethanolamine (PE)	11,304 ± 1916	10,138 ± 1943	13,086 ± 1837	0.55
Sphingomyelin (SM)	3824 ± 213	3722 ± 151	3438 ± 170	0.31
Triacylglycerol (TAG)	177.3 ± 14.3	208.2 ± 18.4	176.5 ± 12.6	0.27

Phosphatidylcholine (PC) levels were significantly reduced in HFD+W mice, while levels of cholesterol ester (CE), ceramide (CER), lysophosphatidylcholine (LPC) and lysophosphatidylethanolamine (LPE) tended to be lower in HFD+W. Different letters denote a significant difference between groups, *p* < 0.05.

**Table 3:** Composition of the purified diet formulations.

Component (g/kg)	LFD	HFD	HFD+Walnut
Casein	210.0	245.0	231.8
L-Cystine	3.0	3.5	3.5
Corn Starch	465	85	85
Maltodextrin	100	115	102
Sucrose	90	200	200
Lard	20.0	195.0	145.6
Soybean Oil	20	30	30
Cellulose	37.2	58.0	58.0
Mineral Mix, AIN-93G-MX (94046)	35	43	43
Calcium Phosphate, dibasic	2.0	3.4	3.4
Vitamin Mix, AIN-93-VX (94047)	15	19	19
Choline Bitartrate	2.75	3.00	3.00
Kcal from CHO, %	69.1	36.2	36.1
Kcal from PRO, %	20.5	19.0	19.0
Kcal from Fat, %	10.4	44.8	44.9
Walnuts, ground	0.0	0.0	75.7
Kcal/g	3.6	4.6	4.6
Catalog number	TD.08806	TD.06415	TD.140817

All purified formulas were produced onsite at Envigo, Inc., Madison, WI, USA.

## Data Availability

All data are freely available from the corresponding author upon reasonable request.

## Supplementary Materials

The following supporting information can be downloaded at: https://www.mdpi.com/article/10.3390/ijms24032314/s1.

## References

[1] Barzilai N., Huffman D.M., Muzumdar R.H., Bartke A. (2012). The critical role of metabolic pathways in aging. Diabetes.

[2] Harrison D.E., Strong R., Alavez S., Astle C.M., DiGiovanni J., Fernandez E., Flurkey K., Garratt M., Gelfond J.A.L., Javors M.A. (2019). Acarbose improves health and lifespan in aging HET3 mice. Aging Cell.

[3] Nadon N.L., Strong R., Miller R.A., Harrison D.E. (2017). NIA Interventions Testing Program: Investigating Putative Aging Intervention Agents in a Genetically Heterogeneous Mouse Model. EBioMedicine.

[4] Harrison D.E., Strong R., Sharp Z.D., Nelson J.F., Astle C.M., Flurkey K., Nadon N.L., Wilkinson J.E., Frenkel K., Carter C.S. (2009). Rapamycin fed late in life extends lifespan in genetically heterogeneous mice. Nature.

[5] Xie K., Neff F., Markert A., Rozman J., Aguilar-Pimentel J.A., Amarie O.V., Becker L., Brommage R., Garrett L., Henzel K.S. (2017). Every-other-day feeding extends lifespan but fails to delay many symptoms of aging in mice. Nat. Commun..

[6] Honjoh S., Yamamoto T., Uno M., Nishida E. (2009). Signalling through RHEB-1 mediates intermittent fasting-induced longevity in C. elegans. Nature.

[7] Newman J.C., Covarrubias A.J., Zhao M., Yu X., Gut P., Ng C.P., Huang Y., Haldar S., Verdin E. (2017). Ketogenic Diet Reduces Midlife Mortality and Improves Memory in Aging Mice. Cell Metab..

[8] Roberts M.N., Wallace M.A., Tomilov A.A., Zhou Z., Marcotte G.R., Tran D., Perez G., Gutierrez-Casado E., Koike S., Knotts T.A. (2017). A Ketogenic Diet Extends Longevity and Healthspan in Adult Mice. Cell Metab..

[9] Solon-Biet S.M., Cogger V.C., Pulpitel T., Wahl D., Clark X., Bagley E., Gregoriou G.C., Senior A.M., Wang Q.-P., Brandon A.E. (2019). Branched chain amino acids impact health and lifespan indirectly via amino acid balance and appetite control. Nat. Metab..

[10] Lee B.C., Kaya A., Ma S., Kim G., Gerashchenko M.V., Yim S.H., Hu Z., Harshman L.G., Gladyshev V.N. (2014). Methionine restriction extends lifespan of Drosophila melanogaster under conditions of low amino-acid status. Nat. Commun..

[11] Richie J.P., Leutzinger Y., Parthasarathy S., Malloy V., Orentreich N., Zimmerman J.A. (1994). Methionine restriction increases blood glutathione and longevity in F344 rats. FASEB J..

[12] Orentreich N., Matias J.R., DeFelice A., Zimmerman J.A. (1993). Low methionine ingestion by rats extends life span. J. Nutr..

[13] Sataranatarajan K., Pharaoh G., Brown J.L., Ranjit R., Piekarz K.M., Street K., Wren J.D., Georgescu C., Kinter C., Kinter M. (2020). Molecular changes in transcription and metabolic pathways underlying muscle atrophy in the CuZnSOD null mouse model of sarcopenia. Geroscience.

[14] Perez V.I., Van Remmen H., Bokov A., Epstein C.J., Vijg J., Richardson A. (2009). The overexpression of major antioxidant enzymes does not extend the lifespan of mice. Aging Cell.

[15] Massie H.R., Aiello V.R., Doherty T.J. (1984). Dietary vitamin C improves the survival of mice. Gerontology.

[16] Ristow M., Zarse K., Oberbach A., Kloting N., Birringer M., Kiehntopf M., Stumvoll M., Kahn C.R., Blüher M. (2009). Antioxidants prevent health-promoting effects of physical exercise in humans. Proc. Natl. Acad. Sci. USA.

[17] Baur J.A., Pearson K.J., Price N.L., Jamieson H.A., Lerin C., Kalra A., Prabhu V.V., Allard J.S., Lopez-Lluch G., Lewis K. (2006). Resveratrol improves health and survival of mice on a high-calorie diet. Nature.

[18] Strong R., Miller R.A., Astle C.M., Baur J.A., de Cabo R., Fernandez E., Guo W., Javors M., Kirkland J.L., Nelson J.F. (2013). Evaluation of resveratrol, green tea extract, curcumin, oxaloacetic acid, and medium-chain triglyceride oil on life span of genetically heterogeneous mice. J. Gerontol. A Biol. Sci. Med. Sci..

[19] Miller R.A., Harrison D.E., Astle C.M., Bogue M.A., Brind J., Fernandez E., Flurkey K., Javors M., Ladiges W., Leeuwenburgh C. (2019). Glycine supplementation extends lifespan of male and female mice. Aging Cell.

[20] Eisenberg T., Abdellatif M., Schroeder S., Primessnig U., Stekovic S., Pendl T., Harger A., Schipke J., Zimmermann A., Schmidt A. (2016). Cardioprotection and lifespan extension by the natural polyamine spermidine. Nat. Med..

[21] Freedman N.D., Park Y., Abnet C.C., Hollenbeck A.R., Sinha R. (2012). Association of coffee drinking with total and cause-specific mortality. N. Engl. J. Med..

[22] Bao Y., Han J., Hu F.B., Giovannucci E.L., Stampfer M.J., Willett W.C., Fuchs C.S. (2013). Association of nut consumption with total and cause-specific mortality. N. Engl. J. Med..

[23] Pan A., Sun Q., Manson J.E., Willett W.C., Hu F.B. (2013). Walnut consumption is associated with lower risk of type 2 diabetes in women. J. Nutr..

[24] Banel D.K., Hu F.B. (2009). Effects of walnut consumption on blood lipids and other cardiovascular risk factors: A meta-analysis and systematic review. Am. J. Clin. Nutr..

[25] Poulose S.M., Miller M.G., Shukitt-Hale B. (2014). Role of walnuts in maintaining brain health with age. J. Nutr..

[26] Hardman W.E. (2014). Walnuts have potential for cancer prevention and treatment in mice. J. Nutr..

[27] Kris-Etherton P.M. (2014). Walnuts decrease risk of cardiovascular disease: A summary of efficacy and biologic mechanisms. J. Nutr..

[28] Anderson K.J., Teuber S.S., Gobeille A., Cremin P., Waterhouse A.L., Steinberg F.M. (2001). Walnut polyphenolics inhibit in vitro human plasma and LDL oxidation. J. Nutr..

[29] Nagel J.M., Brinkoetter M., Magkos F., Liu X., Chamberland J.P., Shah S., Zhou J., Blackburn G., Mantzoros C.S. (2012). Dietary walnuts inhibit colorectal cancer growth in mice by suppressing angiogenesis. Nutrition.

[30] Guan F., Tabrizian T., Novaj A., Nakanishi M., Rosenberg D.W., Huffman D.M. (2018). Dietary Walnuts Protect Against Obesity-Driven Intestinal Stem Cell Decline and Tumorigenesis. Front Nutr..

[31] Miller R.A., Harrison D.E., Astle C.M., Baur J.A., Boyd A.R., de Cabo R., Fernandez E., Flurkey K., Javors M.A., Nelson J.F. (2011). Rapamycin, but not resveratrol or simvastatin, extends life span of genetically heterogeneous mice. J. Gerontol. A Biol. Sci. Med. Sci..

[32] Mao K., Quipildor G.F., Tabrizian T., Novaj A., Guan F., Walters R.O., Delahaye F., Hubbard G.B., Ikeno Y., Ejima K. (2018). Late-life targeting of the IGF-1 receptor improves healthspan and lifespan in female mice. Nat. Commun..

[33] Farias Quipildor G.E., Mao K., Hu Z., Novaj A., Cui M.H., Gulinello M., Branch C.A., Gubbi S., Patel K., Moellering D.R. (2019). Central IGF-1 protects against features of cognitive and sensorimotor decline with aging in male mice. Geroscience.

[34] Sabate J., Fraser G.E., Burke K., Knutsen S.F., Bennett H., Lindsted K.D. (1993). Effects of walnuts on serum lipid levels and blood pressure in normal men. N. Engl. J. Med..

[35] Zambon D., Sabate J., Munoz S., Campero B., Casals E., Merlos M., Laguna J.C., Ros E. (2000). Substituting walnuts for monounsaturated fat improves the serum lipid profile of hypercholesterolemic men and women. A randomized crossover trial. Ann. Intern. Med..

[36] Ros E., Nunez I., Perez-Heras A., Serra M., Gilabert R., Casals E., Deulofeu R., Ros E. (2004). A walnut diet improves endothelial function in hypercholesterolemic subjects: A randomized crossover trial. Circulation.

[37] Halvorsen B.L., Carlsen M.H., Phillips K.M., Bohn S.K., Holte K., Jacobs D.R., Blomhoff R. (2006). Content of redox-active compounds (ie, antioxidants) in foods consumed in the United States. Am. J. Clin. Nutr..

[38] Nakanishi M., Chen Y., Qendro V., Miyamoto S., Weinstock E., Weinstock G.M., Rosenberg D.W. (2016). Effects of Walnut Consumption on Colon Carcinogenesis and Microbial Community Structure. Cancer Prev. Res. (Phila).

[39] Tsoukas M.A., Ko B.J., Witte T.R., Dincer F., Hardman W.E., Mantzoros C.S. (2015). Dietary walnut suppression of colorectal cancer in mice: Mediation by miRNA patterns and fatty acid incorporation. J. Nutr. Biochem..

[40] Hardman W.E., Ion G., Akinsete J.A., Witte T.R. (2011). Dietary walnut suppressed mammary gland tumorigenesis in the C(3)1 TAg mouse. Nutr. Cancer.

[41] Nakanishi M., Matz A., Klemashevich C., Rosenberg D.W. (2019). Dietary Walnut Supplementation Alters Mucosal Metabolite Profiles During DSS-Induced Colonic Ulceration. Nutrients.

[42] Pandareesh M.D., Chauhan V., Chauhan A. (2018). Walnut Supplementation in the Diet Reduces Oxidative Damage and Improves Antioxidant Status in Transgenic Mouse Model of Alzheimer’s Disease. J. Alzheimers Dis..

[43] Muthaiyah B., Essa M.M., Lee M., Chauhan V., Kaur K., Chauhan A. (2014). Dietary supplementation of walnuts improves memory deficits and learning skills in transgenic mouse model of Alzheimer’s disease. J. Alzheimers Dis..

[44] Sala-Vila A., Valls-Pedret C., Rajaram S., Coll-Padros N., Cofan M., Serra-Mir M., Pérez-Heras A.M., Roth I., Freitas-Simoes T.M., Doménech M. (2020). Effect of a 2-year diet intervention with walnuts on cognitive decline. The Walnuts And Healthy Aging (WAHA) study: A randomized controlled trial. Am. J. Clin. Nutr..

[45] Pribis P., Bailey R.N., Russell A.A., Kilsby M.A., Hernandez M., Craig W.J., Grajales T., Shavlik D.J., Sabatè J. (2012). Effects of walnut consumption on cognitive performance in young adults. Br. J. Nutr..

[46] Bishop N.J., Zuniga K.E. (2020). Investigating walnut consumption and cognitive trajectories in a representative sample of older US adults. Public Health Nutr..

[47] Choi Y., Abdelmegeed M.A., Song B.J. (2016). Preventive effects of dietary walnuts on high-fat-induced hepatic fat accumulation, oxidative stress and apoptosis in mice. J. Nutr. Biochem..

[48] Smith D.L., Yang Y., Nagy T.R., Patki A., Vasselli J.R., Zhang Y., Dickinson S.L., Allison D.B. (2018). Weight Cycling Increases Longevity Compared with Sustained Obesity in Mice. Obesity (Silver Spring).

[49] Augenlicht L. (2014). Hidden effects of mouse chow. Science.

[50] List E.O., Berryman D.E., Wright-Piekarski J., Jara A., Funk K., Kopchick J.J. (2013). The effects of weight cycling on lifespan in male C57BL/6J mice. Int. J. Obes. (Lond).

[51] Surra J.C., Barranquero C., Torcal M.P., Orman I., Segovia J.C., Guillen N., Navarro M.A., Arnal C., Osada J. (2013). In comparison with palm oil, dietary nut supplementation delays the progression of atherosclerotic lesions in female apoE-deficient mice. Br. J. Nutr..

[52] Wang S., Zheng L., Zhao T., Zhang Q., Liu Y., Sun B., Su G., Zhao M. (2020). Inhibitory Effects of Walnut (Juglans regia) Peptides on Neuroinflammation and Oxidative Stress in Lipopolysaccharide-Induced Cognitive Impairment Mice. J. Agric. Food Chem..

[53] Poulose S.M., Bielinski D.F., Shukitt-Hale B. (2013). Walnut diet reduces accumulation of polyubiquitinated proteins and inflammation in the brain of aged rats. J. Nutr. Biochem..

[54] Moran J., Garrido P., Alonso A., Cabello E., Gonzalez C. (2013). 17beta-Estradiol and genistein acute treatments improve some cerebral cortex homeostasis aspects deteriorated by aging in female rats. Exp. Gerontol..

[55] Sumien N., Chaudhari K., Sidhu A., Forster M.J. (2013). Does phytoestrogen supplementation affect cognition differentially in males and females?. Brain Res..

[56] Esselun C., Dilberger B., Silaidos C.V., Koch E., Schebb N.H., Eckert G.P. (2020). A Walnut Diet in Combination with Enriched Environment Improves Cognitive Function and Affects Lipid Metabolites in Brain and Liver of Aged NMRI Mice. Neuromolecular Med..

[57] Chauhan A., Chauhan V. (2020). Beneficial Effects of Walnuts on Cognition and Brain Health. Nutrients.

[58] Nogales-Bueno J., Baca-Bocanegra B., Hernandez-Hierro J.M., Garcia R., Barroso J.M., Heredia F.J., Rato A.E. (2021). Assessment of Total Fat and Fatty Acids in Walnuts Using Near-Infrared Hyperspectral Imaging. Front. Plant Sci..

[59] Nakamura M.T., Nara T.Y. (2003). Essential fatty acid synthesis and its regulation in mammals. Prostaglandins Leukot Essent Fat. Acids.

[60] Brenna J.T., Salem N., Sinclair A.J., Cunnane S.C. (2009). International Society for the Study of Fatty A, Lipids I. alpha-Linolenic acid supplementation and conversion to n-3 long-chain polyunsaturated fatty acids in humans. Prostaglandins Leukot Essent Fat. Acids.

[61] Fu Z., Sinclair A.J. (2000). Increased alpha-linolenic acid intake increases tissue alpha-linolenic acid content and apparent oxidation with little effect on tissue docosahexaenoic acid in the guinea pig. Lipids.

[62] Qu M.H., Yang X., Wang Y., Tang Q., Han H., Wang J., Wang G.-D., Xue C., Gao Z. (2016). Docosahexaenoic Acid-Phosphatidylcholine Improves Cognitive Deficits in an Abeta23-35-Induced Alzheimer’s Disease Rat Model. Curr. Top. Med. Chem..

[63] Oliveira T.G., Chan R.B., Bravo F.V., Miranda A., Silva R.R., Zhou B., Marques F., Pinto V., Cerqueira J.J., Di Paolo G. (2016). The impact of chronic stress on the rat brain lipidome. Mol. Psychiatry.

[64] Harrison D.E., Strong R., Reifsnyder P., Kumar N., Fernandez E., Flurkey K., Javors M.A., Lopez-Cruzan M., Macchiarini F., Nelson J.F. (2021). 17-a-estradiol late in life extends lifespan in aging UM-HET3 male mice; nicotinamide riboside and three other drugs do not affect lifespan in either sex. Aging Cell.

[65] Miller R.A., Harrison D.E., Allison D.B., Bogue M., Debarba L., Diaz V., Fernandez E., Galecki A., Garvey W.T., Jayarathne H. (2020). Canagliflozin extends life span in genetically heterogeneous male but not female mice. JCI Insight.

[66] Weiss R., Fernandez E., Liu Y., Strong R., Salmon A.B. (2018). Metformin reduces glucose intolerance caused by rapamycin treatment in genetically heterogeneous female mice. Aging (Albany NY).

[67] Harrison D.E., Strong R., Allison D.B., Ames B.N., Astle C.M., Atamna H., Fernandez E., Flurkey K., Javors M.A., Nadon N.L. (2014). Acarbose, 17-alpha-estradiol, and nordihydroguaiaretic acid extend mouse lifespan preferentially in males. Aging Cell.

[68] Huffman D.M., Augenlicht L.H., Zhang X., Lofrese J.J., Atzmon G., Chamberland J.P., Mantzoros C.S. (2013). Abdominal obesity, independent from caloric intake, accounts for the development of intestinal tumors in Apc(1638N/+) female mice. Cancer Prev. Res. (Phila).

[69] Walters R.O., Arias E., Diaz A., Burgos E.S., Guan F., Tiano S., Mao K., Green C.L., Qiu Y., Shah H. (2018). Sarcosine Is Uniquely Modulated by Aging and Dietary Restriction in Rodents and Humans. Cell Rep..

[70] Whitehead J.C., Hildebrand B.A., Sun M., Rockwood M.R., Rose R.A., Rockwood K., Howlett S.E. (2014). A clinical frailty index in aging mice: Comparisons with frailty index data in humans. J. Gerontol. A Biol. Sci. Med. Sci..

[71] Engel M.G., Smith J., Mao K., Quipildor G.F., Cui M.H., Gulinello M., Branch C.A., Gandy S.E., Huffman D.M. (2022). Evidence for preserved insulin responsiveness in the aging rat brain. Geroscience.

[72] Farias Quipildor G., Mao K., Beltran P.J., Barzilai N., Huffman D.M. (2021). Modulation of Glucose Production by Central Insulin Requires IGF-1 Receptors in AgRP Neurons. Diabetes.

[73] Tabrizian T., Wang D., Guan F., Hu Z., Beck A.P., Delahaye F., Huffman D.M. (2017). Apc inactivation, but not obesity, synergizes with Pten deficiency to drive intestinal stem cell-derived tumorigenesis. Endocr. Relat. Cancer.

[74] Dobin A., Davis C.A., Schlesinger F., Drenkow J., Zaleski C., Jha S., Batut P., Chaisson M., Gingeras T.R. (2013). STAR: Ultrafast universal RNA-seq aligner. Bioinformatics.

[75] Liao Y., Smyth G.K., Shi W. (2014). featureCounts: An efficient general purpose program for assigning sequence reads to genomic features. Bioinformatics.

[76] Love M.I., Huber W., Anders S. (2014). Moderated estimation of fold change and dispersion for RNA-seq data with DESeq2. Genome Biol..

[77] Patro R., Duggal G., Love M.I., Irizarry R.A., Kingsford C. (2017). Salmon provides fast and bias-aware quantification of transcript expression. Nat. Methods.

[78] Miller N.J., Rice-Evans C., Davies M.J., Gopinathan V., Milner A. (1993). A novel method for measuring antioxidant capacity and its application to monitoring the antioxidant status in premature neonates. Clin. Sci. (Lond).

[79] Rice-Evans C.A. (2000). Measurement of total antioxidant activity as a marker of antioxidant status in vivo: Procedures and limitations. Free Radic. Res..

[80] Ghiselli A., Serafini M., Natella F., Scaccini C. (2000). Total antioxidant capacity as a tool to assess redox status: Critical view and experimental data. Free Radic. Biol. Med..

[81] Rice-Evans C., Miller N.J. (1994). Total antioxidant status in plasma and body fluids. Methods Enzymol..

[82] Hanson A.J., Banks W.A., Bettcher L.F., Pepin R., Raftery D., Craft S. (2019). Cerebrospinal fluid lipidomics: Effects of an intravenous triglyceride infusion and apoE status. Metabolomics.

