# Supplementary material for: Dietary Walnuts Preserve Aspects of Health Span and Alter the Hippocampal Lipidome in Aged High-Fat Diet-Fed Mice
Source: Int J Mol Sci. 2023 Jan 24;24(3):2314. doi: 10.3390/ijms24032314 (PMC9916809; doi:10.3390/ijms24032314)
Supplement: Supplementary file 1 [file ijms-24-02314-s001.zip › ijms-2159067-supplementary.pdf]

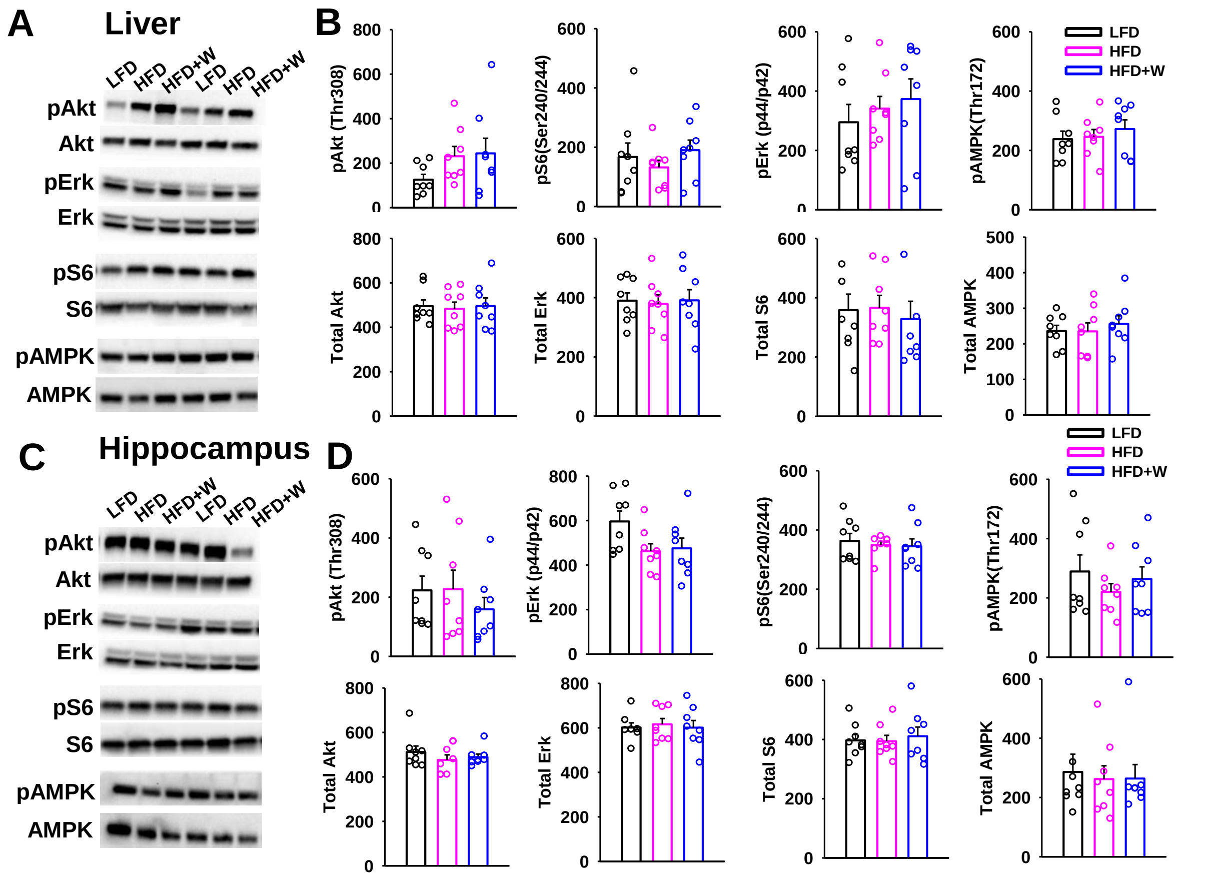

Supplementary Figure S1. Effect of dietary walnuts on metabolic signaling mediators in liver and hippocampus of older CB6F1 male mice. Several markers of relevant metabolic pathways in liver and hippocampus by Western blotting, including Akt, Erk, S6 and AMPK. (A-B) In liver, a non-significant increase in pAkt in high-fat fed groups was observed, but phosphorylated and total levels of Akt, S6, Erk and AMPK were otherwise unaffected by diet under basal conditions (C-D) In hippocampus, a slight, albeit non-significant decrease in pErk was observed in both HFD and HFD+W animals, but phosphorylated and total levels of Akt, S6, Erk and AMPK were no significantly different among groups. Dot plots overlaid on bar graphs represent individual data points. No significant differences were observed among groups for any marker assessed in liver or hippocampus.

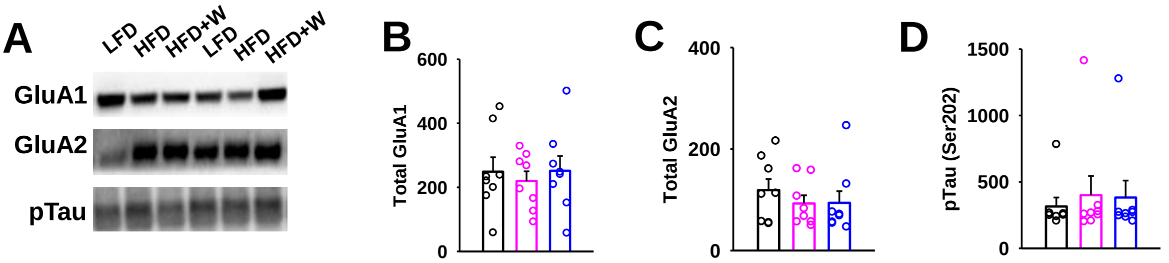

Supplementary Figure S2. Markers of neuronal subunits and Tau phosphorylation in hippocampus of aged mice on different diets. (A-C) Western blotting was performed for the AMPA receptor subunits GluA1 and GluA2, but no significant differences were observed among groups. (A,D) Likewise, while spuriously high pTau levels were observed in some mice, no significant difference was observed in this marker among groups. There were no significant differences among groups.

Novaj *et al.* Supplementary Figure S3

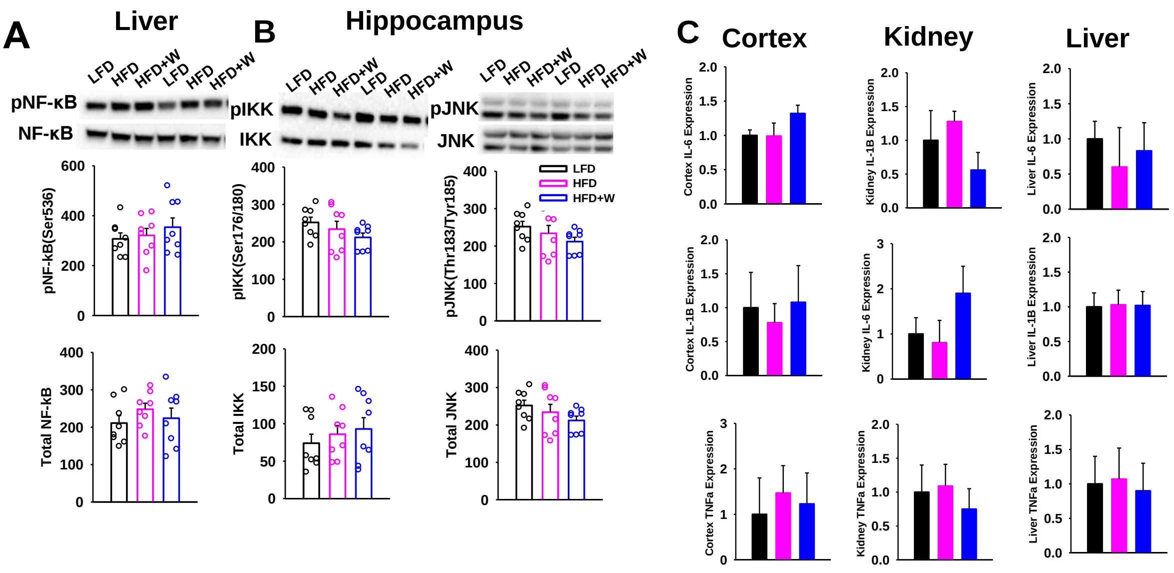

Supplementary Figure S3. Markers (A-B) The effect of diets on the NF-κB signaling pathway and stress-activated protein kinase, JNK, in liver and hippocampal tissue were assessed by Western blotting. In liver and hippocampus, respectively, indicators of NF-κB or JNK activation did not differ among groups. (C) We also performed qPCR for inflammatory markers in liver, kidney and cortex, including *IL-1β*, *TNF-α*, or *IL-6* (n=8-10 per group). Expression was normalized to *PPIA* expression and calculated as fold change versus LFD animals. In liver and cortex, no significant differences were observed among groups in these markers. Moreover, while a numerical reduction in *IL-1β* and increase in *IL-6* was observed in kidney from HFD+W mice, these changes were likewise not significant. Bar graphs represent mean±SE. Dot plots overlaid on bar graphs represent individual data points.

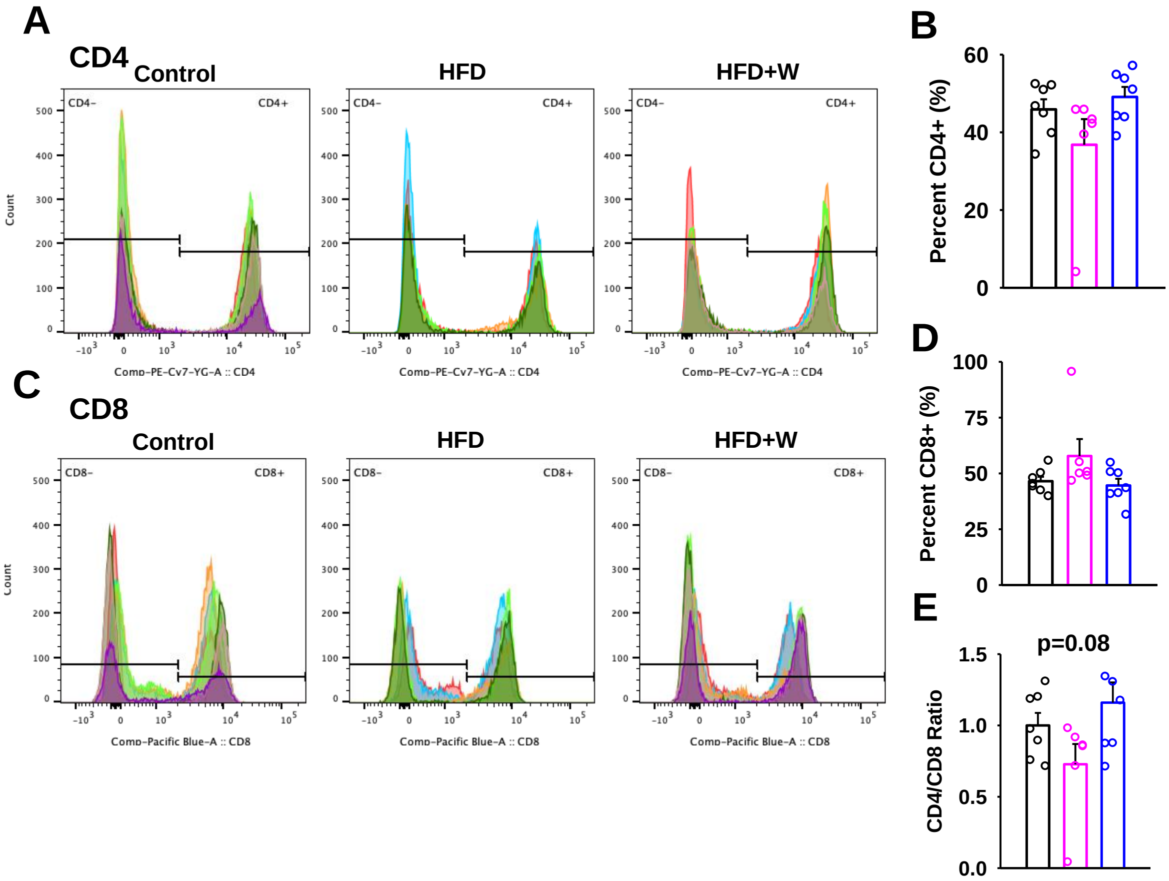

Supplementary Figure S4. T cell immunophenotyping from spleen in aged male mice on different diets. (A-B) CD4+ T cells isolated from spleen tended to be lower in HFD animals, but preserved in HFD+W mice, relative to LFD. (C-D) Meanwhile, the proportion of CD8+ T cells tended to be elevated in HFD control mice. (E) Collectively, the CD4/CD8 T cell ration tended to be lower in HFD animals (p=0.08). Bar graphs represent mean±SE. Dot plots overlaid on bar graphs represent individual data points.
